# Supplementary material for: Associations of healthy lifestyle and socioeconomic status with cognitive function in U.S. older adults
Source: Sci Rep. 2023 May 9;13:7513. doi: 10.1038/s41598-023-34648-0 (PMC10170128; doi:10.1038/s41598-023-34648-0)
Supplement: Supplementary file 1 — Supplementary Information. [file 41598_2023_34648_MOESM1_ESM.docx]

**Online-Only Supplemental Material**

**Associations of Healthy Lifestyle and Socioeconomic Status with Cognitive Function in U.S. Older Adults**

*Xin Wang, Kelly M. Bakulski, Henry L. Paulson, Roger L. Albin, Sung Kyun Park*

**Table S1** The healthy eating index-2015 components and scoring standards.^*^

| Component | Max points | Standard for maximum score | Standard for minimum score |
| --- | --- | --- | --- |
| Adequacy |  |  |  |
| Total fruits (including fruit juice) | 5 | ≥0.8 cup equiv. per 1,000 kcal | No Fruits |
| Whole fruits (except fruit juice) | 5 | ≥0.4 cup equiv. per 1,000 kcal | No Whole Fruits |
| Total vegetables | 5 | ≥1.1 cup equiv. per 1,000 kcal | No Vegetables |
| Greens and beans | 5 | ≥0.2 cup equiv. per 1,000 kcal | No Greens and Beans |
| Whole grains | 10 | ≥1.5 oz equiv. per 1,000 kcal | No Whole Grains |
| Dairy | 10 | ≥1.3 cup equiv. per 1,000 kcal | No Dairy |
| Total protein foods | 5 | ≥2.5 oz equiv. per 1,000 kcal | No Protein Foods |
| Seafood and plant proteins | 5 | ≥0.8 oz equiv. per 1,000 kcal | No Seafood or Plant Proteins |
| Fatty acids^†^ | 10 | (PUFAs + MUFAs)/SFAs ≥2.5 | (PUFAs + MUFAs)/SFAs ≤1.2 |
| Moderation |  |  |  |
| Refined grains | 10 | ≤1.8 oz equiv. per 1,000 kcal | ≥4.3 oz equiv. per 1,000 kcal |
| Sodium | 10 | ≤1.1 gram per 1,000 kcal | ≥2.0 grams per 1,000 kcal |
| Added sugars | 10 | ≤6.5% of energy | ≥26% of energy |
| Saturated fats | 10 | ≤8% of energy | ≥16% of energy |

* The table was derived from Krebs-Smith SM, Pannucci TRE, Subar AF, et al. Update of the Healthy Eating Index: HEI-2015. J Acad Nutr Diet. Elsevier; 2018;118:1591–1602.

† Fatty acids include polyunsaturated fatty acids (PUFAs), monounsaturated fatty acids (MUFAs), and saturated fatty acids (SFAs).

**Table S2** The results from the latent class analysis for composite socioeconomic status (SES).

|  | High SES | Medium SES | Low SES |
| --- | --- | --- | --- |
|  | Probability | Probability | Probability |
| Income-to-poverty ratio |  |  |  |
| Low (≤1) | 0.03 | 0.01 | 0.46 |
| Medium (>1 and <4) | 0.47 | 0.81 | 0.54 |
| High (≥4) | 0.5 | 0.18 | 0 |
| Education |  |  |  |
| <high school | 0 | 0.29 | 0.46 |
| High school | 0.11 | 0.38 | 0.24 |
| College or higher | 0.89 | 0.33 | 0.3 |
| Occupation^*^ |  |  |  |
| Blue collar | 0.06 | 0.66 | 0.65 |
| White collar | 0.93 | 0.31 | 0.29 |
| Not in labor | 0.01 | 0.03 | 0.05 |
| Unemployed | 0 | 0 | 0.01 |
| Health insurance |  |  |  |
| Uninsured | 0.03 | 0.03 | 0.18 |
| Government insurance | 0.24 | 0.35 | 0.65 |
| Private insurance | 0.29 | 0.21 | 0.06 |
| Both government and private | 0.44 | 0.41 | 0.11 |
| Food security |  |  |  |
| Having food insecurity | 0.03 | 0.08 | 0.54 |
| Full food security | 0.97 | 0.92 | 0.46 |

^*^ Blue collar occupations included service, farming/forestry/fishing, precision production/craft/repair, and operator/fabricator/laborer occupations. White collar occupations included managerial/professional specialty, and technical/sales/administrative support occupations. Not in labor force included retired persons, homemakers, and students.

**Table S3** Survey-weighted differences and 95% confidence intervals (95% CIs)^*^ in z-scores of four cognitive tests with socioeconomic status and healthy lifestyle from the U.S. National Health and Nutrition Examination Survey 2011-2014.

|  | CERAD^†^ Word List learning | CERAD delayed recall | Animal Fluency | Digit Symbol Substitution Test |
| --- | --- | --- | --- | --- |
|  | Change (95% CI) | Change (95% CI) | Change (95% CI) | Change (95% CI) |
| Healthy lifestyle score |  |  |  |  |
| 0 or 1 healthy behavior | REF | REF | REF | REF |
| 2 healthy behaviors | 0.06 (-0.13, 0.25) | -0.03 (-0.16, 0.11) | 0.02 (-0.12, 0.15) | 0.07 (-0.08, 0.22) |
| 3 or 4 healthy behaviors | 0.12 (-0.05, 0.29) | 0.09 (-0.05, 0.23) | -0.03 (-0.18, 0.12) | 0.12 (-0.02, 0.25) |
| Socioeconomic status |  |  |  |  |
| Low SES | REF | REF | REF | REF |
| Medium SES | 0.07 (-0.11, 0.25) | -0.007 (-0.16, 0.10) | 0.13 (-0.07, 0.33) | 0.34 (0.16, 0.51)** |
| High SES | 0.45 (0.28, 0.62)*** | -0.03 (-0.16, 0.10) | 0.36 (0.20, 0.53)*** | 0.72 (0.55, 0.89)*** |

^*^ Model was adjusted for age, sex, race/ethnicity, marital status, and birthplace.

^†^ Consortium to Establish a Registry for Alzheimer’s Disease (CERAD).

*P<0.05, **P<0.01, ***P<0.0001

**Table S4** Survey-weighted differences and 95% confidence intervals (95% CIs)^*^ in cognitive composite z-score with socioeconomic status and weighted healthy lifestyle from the U.S. National Health and Nutrition Examination Survey 2011-2014.

|  | Change (95% CI) |
| --- | --- |
| Weighted healthy lifestyle score |  |
| Tertile 1 | REF |
| Tertile 2 | 0.01 (-0.06, 0.09) |
| Tertile 3 | 0.08 (0.03, 0.12)** |
| Socioeconomic status |  |
| Low SES | REF |
| Medium SES | 0.13 (0.03, 0.23)* |
| High SES | 0.37 (0.28, 0.46)*** |

^*^ Model was adjusted for age, sex, race/ethnicity, marital status, and birthplace.

*P<0.05, **P<0.01, ***P<0.0001

**Table S5** Survey-weighted differences and 95% confidence intervals (95% CIs)^*^ in cognitive composite z-score with socioeconomic status and healthy lifestyle with an additional adjustment for body mass index from the U.S. National Health and Nutrition Examination Survey 2011-2014.

|  | Change (95% CI) |
| --- | --- |
| Healthy lifestyle score |  |
| Tertile 1 | REF |
| Tertile 2 | 0.03 (-0.05, 0.11) |
| Tertile 3 | 0.07 (0.005, 0.14)* |
| Socioeconomic status |  |
| Low SES | REF |
| Medium SES | 0.13 (0.03, 0.24)* |
| High SES | 0.37 (0.29, 0.46)*** |

^*^ Model was adjusted for age, sex, race/ethnicity, marital status, birthplace, and body mass index.

*P<0.05, **P<0.01, ***P<0.0001

**Table S6** Survey-weighted differences and 95% confidence intervals (95% CIs)^*^ in cognitive composite z-score with socioeconomic status and healthy lifestyle from the U.S. National Health and Nutrition Examination Survey 2011-2014 after excluding 283 participants with cardiovascular disease or stroke.

|  | Change (95% CI) |
| --- | --- |
| Healthy lifestyle score |  |
| 0 or 1 healthy behavior | REF |
| 2 healthy behaviors | 0.02 (-0.06, 0.10) |
| 3 or 4 healthy behaviors | 0.09 (0.01, 0.17)* |
| Socioeconomic status |  |
| Low SES | REF |
| Medium SES | 0.13 (0.04, 0.22)** |
| High SES | 0.41 (0.32, 0.50)*** |

^*^ Model was adjusted for age, sex, race/ethnicity, marital status, and birthplace.

*P<0.05, **P<0.01, ***P<0.0001

**Table S7** Survey-weighted differences and 95% confidence intervals (95% CIs)^*^ in cognitive composite z-score with an additional interaction term^†^ between socioeconomic status (SES) and healthy lifestyle from the U.S. National Health and Nutrition Examination Survey after excluding 283 participants with cardiovascular disease or stroke.

|  | Socioeconomic status | | |
| --- | --- | --- | --- |
| Healthy lifestyle score | Low SES | Medium SES | High SES |
| 0 or 1 healthy behavior | REF | 0.22 (0.06, 0.38)** | 0.32 (0.16, 0.48)** |
| 2 healthy behaviors | -0.01 (-0.17, 0.16) | 0.04 (-0.11, 0.19) | 0.45 (0.32, 0.57)*** |
| 3 or 4 healthy behaviors | 0.05 (-0.08, 0.19) | 0.19 (0.04, 0.35)* | 0.49 (0.36, 0.60)*** |

* Model was adjusted for age, sex, race/ethnicity, marital status, and birthplace.

† P for interaction between SES and healthy lifestyle was 0.09.

*P<0.05, **P<0.01, ***P<0.0001

**Table S8** Survey-weighted differences and 95% confidence intervals (95% CIs) in cognitive composite z-score from linear regression models with 20 multiple imputations from the U.S. National Health and Nutrition Examination Survey 2011-2014.

|  | Model 1 | Model 2 | Model 3 |
| --- | --- | --- | --- |
|  | Change (95% CI) | Change (95% CI) | Change (95% CI) |
| Lifestyle factors |  |  |  |
| Smoking status |  |  |  |
| Former or current smoker | REF | − | REF |
| Never smoker | 0.08 (0.01, 0.14)* | − | 0.02 (-0.03, 0.08) |
| Alcohol consumption^*^ |  |  |  |
| High | REF | − | REF |
| Low or moderate | -0.14 (-0.16, -0.12)** | − | -0.07 (-0.16, 0.007) |
| Physical activity |  |  |  |
| Not healthy | REF | − | REF |
| Healthy | 0.05 (-0.01, 0.11) | − | 0.05 (0, 0.10)* |
| Healthy eating index |  |  |  |
| Not healthy | REF | − | REF |
| Healthy | 0.12 (0.11, 0.13)*** | − | 0.06 (0.02, 0.10)** |
| Socioeconomic status |  |  |  |
| Income-to-povertyratio |  |  |  |
| ≤1 | − | REF | REF |
| >1 and <4 | − | 0.08 (0.005, 0.15)* | 0.07 (0.0007, 0.15)* |
| ≥4 | − | 0.17 (0.10, 0.25)*** | 0.16 (0.08, 0.24)*** |
| Education |  |  |  |
| <High school | − | REF | REF |
| High school or equivalent | − | 0.12 (0.07, 0.17)*** | 0.11 (0.06, 0.17)*** |
| >High school | − | 0.32 (0.25, 0.38)*** | 0.30 (0.23, 0.37)*** |
| Insurance |  |  |  |
| Uninsured | − | REF | REF |
| Covered by government | − | 0.06 (-0.03, 0.14) | 0.06 (-0.03, 0.15) |
| Covered by private | − | 0.07 (-0.02, 0.17) | 0.06 (-0.03, 0.16) |
| Covered by both | − | 0.06 (-0.02, 0.14) | 0.06 (-0.03, 0.14) |
| Food security |  |  |  |
| Having food insecurity | − | REF | REF |
| Full food security | − | 0.09 (0.02, 0.17)* | 0.09 (0.02, 0.16)* |
| Occupation |  |  |  |
| Blue collar^†^ | − | REF | REF |
| White collar^‡^ | − | 0.18 (0.12, 0.24)*** | 0.17 (0.11, 0.23)*** |
| Not in labor force^§^ | − | 0.06 (-0.05, 0.16) | 0.05 (-0.06, 0.16) |
| Unemployed | − | 0.27 (0.04, 0.49)* | 0.28 (0.05, 0.51)* |

* Low alcohol consumption was defined as daily consumption of one drink or fewer for women and two drinkers or fewer for men, and otherwise coded as moderate or high consumption.

† Blue collar occupations included service, farming/forestry/fishing, precision production/craft/repair, and operator/fabricator/laborer occupations.

‡White collar occupations included managerial/professional specialty, and technical/sales/administrative support occupations.

§ Not in labor force included retired persons, homemakers, and students.

*P<0.05, **P<0.01, ***P<0.0001


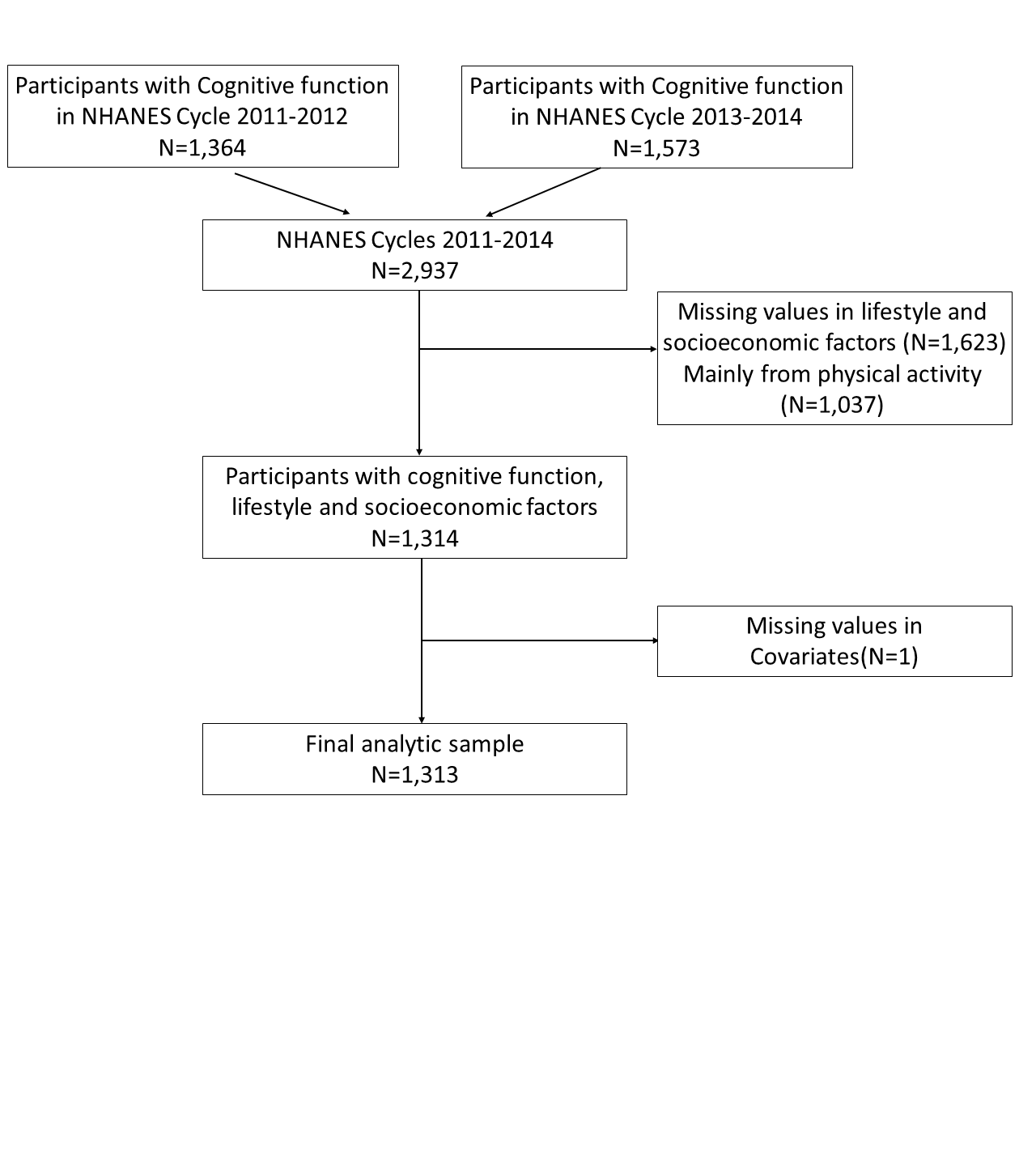


**Figure S1** The flowchart of participant selection in the present study.
